# Role of Dietary Intake and Serum 25(OH)D on the Effects of a Multicomponent Exercise Program on Bone Mass and Structure of Frail and Pre-Frail Older Adults

**DOI:** 10.3390/nu12103016

**Published:** 2020-10-01

**Authors:** Ana Moradell, David Navarrete-Villanueva, Ángel I. Fernández-García, Jorge Marín-Puyalto, Alejandro Gómez-Bruton, Raquel Pedrero-Chamizo, Jorge Pérez-Gómez, Ignacio Ara, Jose A. Casajus, Alba Gómez-Cabello, Germán Vicente-Rodríguez

**Affiliations:** 1 GENUD (Growth, Exercise, NUtrition and Development) Research Group, Universidad de Zaragoza, 50009 Zaragoza, Spain; amoradell@unizar.es (A.M.); dnavarrete@unizar.es (D.N.-V.); angelivanfg@unizar.es (Á.I.F.-G.); jmarinp@unizar.es (J.M.-P.); bruton@unizar.es (A.G.-B.); joseant@unizar.es (J.A.C.); agomez@unizar.es (A.G.-C.); 2 Agrifood Research and Technology Centre of Aragón -IA2-, CITA-Universidad de Zaragoza, 50009 Zaragoza, Spain; 3 Exercise and Health in Special Population Spanish Research Net (EXERNET), 50009 Zaragoza, Spain; raquel.pedrero@upm.es; 4 Faculty of Health and Sport Science FCSD, Department of Physiatry and Nursing, University of Zaragoza, 50009 Zaragoza, Spain; 5 Faculty of Health, Department of Physiatry and Nursing, University of Zaragoza, 50009 Zaragoza, Spain; 6 ImFINE Research Group, Department of Health and Human Performance, Faculty of Physical Activity and Sport Sciences-INEF, Polytechnic University of Madrid, 28040 Madrid, Spain; 7 HEME Research Group, University of Extremadura, 10003 Cáceres, Spain; jorgepg100@gmail.com; 8 GENUD Toledo Research Group, University of Castilla-La Mancha, 45071 Toledo, Spain; ignacio.ara@uclm.es; 9 CIBER of Frailty and Healthy Aging (CIBERFES), 28029 Madrid, Spain; 10 Biomedical Research Net in Physiopatology, Obesity and Nutricition (CIBERObn), 28029 Madrid, Spain; 11 Defense University Center, 50090 Zaragoza, Spain

**Keywords:** osteoporosis, physical activity, polyunsaturated fatty acids, vitamin D, alcohol, frailty

## Abstract

The multicomponent training (MCT) effect on bone health in frail and pre-frail elders, which is influenced by dietary intake, is still unknown. The objective of this non-randomized intervention trial was to assess the effects of a 6-month MCT on bone structure in frail and pre-frail elders, and to analyse the influence of dietary intake and serum vitamin D (25(OH)D) in these changes. Thirty MCT (TRAIN) and sixteen controls (CON), frail and pre-frail completed the information required for this study. Peripheral quantitative computed tomography measurements were taken at 4% and 38% of the tibia length and dietary intake was registered. The 25(OH)D values were obtained from blood samples. Analyses of covariance (ANCOVA) for repeated measures showed significant decreases for CON in total bone mineral content at 38% of tibia length. One factor ANOVAs showed smaller decreases in bone mineral density and cortical thickness percentage of change in TRAIN compared to CON. Linear regression analyses were performed to study the influence of nutrients and 25(OH)D on bone changes. Alcohol showed a negative influence on fracture index changes, while polyunsaturated fatty acid and vitamin A showed a positive association with some bone variables. The 25(OH)D only affected positively the cortical bone mineral density. In conclusion, our MCT seems to slow down some of the bone detriments associated with ageing in frail and pre-frail older adults, with alcohol showing a negative effect on the bone and apparent limited effect of nutrients and serum 25(OH)D on training related changes.

## 1. Introduction

Frailty is a clinical condition defined by the World Health Organization as “a progressive age-related decline in physiological systems that results in decreased reserves of intrinsic capacity, which confers extreme vulnerability to stressors and increases the risk of a range of adverse health outcomes” [1]. Due to its reversible nature, especially at early stages during pre-frailty [2], researchers are still looking for the best treatment to mitigate frailty and reduce its association with morbility and mortality [3,4]. In this line, evidence points out multicomponent training (MCT) programs as one of the most effective interventions in this population, especially to improve physical function [5]. However, less is known about its effects on other bodily impairments associated with ageing, such as the impairment of bone mass and structure.

The bone is a dynamic tissue that is continuously remodelling across the lifespan [6]. A gradual decline is produced with ageing, increasing the risk of osteoporosis, which is defined as a skeletal disease characterized by a decrease in bone mass and density, with a following increase in bone fragility and risk of fractures [7]. As this critical-age related disorder is strongly associated with pre-frail and frail elderly [8], identifying strategies to improve bone health in this population is of great relevance.

In this regard, evidence suggests that past physical activity has beneficial effects on bone health in older adults [9]. When exercise is practiced during adult- and elder-hood, the bone can be preserved and even improved [10,11]. Specific training programmes, such as those focused on strength, are beneficial for bone maintenance and improvement [10]. In relation to MCT, which is one of the most frequent types of training in older people, more evidence is needed to identify the optimal protocol for improving body composition [12] and specifically bone health. Further, to the authors’ knowledge, there is no evidence regarding the effects of MCT on bone related variables in frail and pre-frail elders.

Additionally, dietary intake may influence the effects of exercise on bone; however, not all research considers these variables. There is robust evidence regarding a positive contribution of calcium and vitamin D in bone health, while other dietary components such as proteins, polyunsaturated fatty acids (PUFA), or alcohol are still under discussion [13]. Specifically, vitamin D seems to play a critical role not only in bone health but also in different aspects of ageing and frailty [14], and it has been related to several diseases [15]. In addition, its deficiency has been related to several diseases and it shows a modest prevalence even in those sunnier Europe countries such as Spain [16,17]. Therefore, it has become important to study the associations of the serum level of vitamin D with health parameters in frail and pre-frail adults. Other dietary micronutrients, such as phosphorus and vitamin A have been reported as necessary for bone metabolism; however, when they are consumed in excess they could induce bone loss [13,18]. For these reasons, more contributions to the literature about dietary intake of nutrients as well as serum levels of 25-hydroxyvitamin D (25(OH)D) would be useful to establish clear conclusions about their mediation in the bone changes observed due to exercise in older adults.

Thus, the main objectives of the study were: (1) To describe the effects of a 6-month MCT on bone mass and structure of frail and pre-frail older adults, and (2) to study the contribution of the main bone-related nutrients and serum 25(OH)D to the changes expected during the training period.

## 2. Materials and Methods

### 2.1. Study Design and Participants

**Figure 1:**
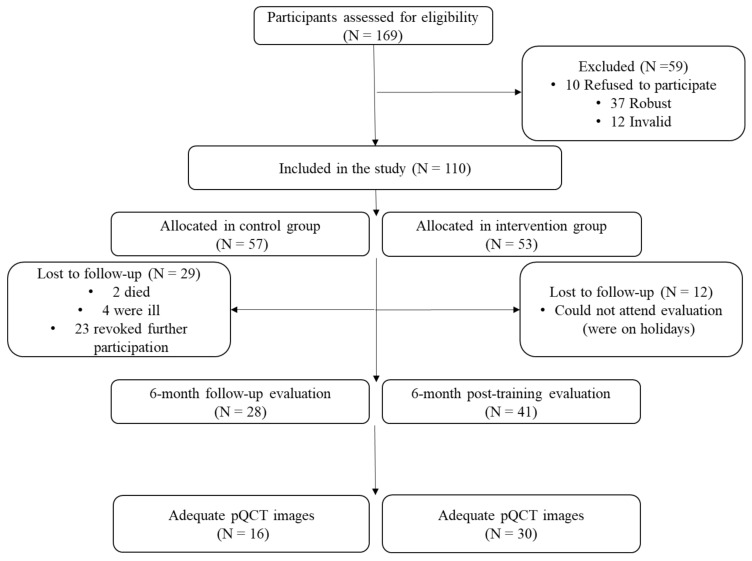
Diagram flow chart of participants. pQCT: Peripheral quantitative computed tomography.

This control trial study was carried out between 2018 and 2020 within the framework of the EXERNET-Elder 3.0 project, which developed a MCT with the main aim of improving physical function in frail and pre-frail older adults. Participants were recruited from four health care centres and three nursing homes from the city of Zaragoza, Spain. People above 65 years categorized as frail or pre-frail according to their functionality measured by the Short Physical Performance Battery [19] were included in the study. Those who had cancer and/or dementia were excluded. In total, 169 elders were initially derived from the centres mentioned above, and finally 110 met the inclusion criteria and were included in the sample as shown in Figure 1.

Participants were then allocated by convenience into two groups, taking into account their preferences and availability: The control group (CON) and training group (TRAIN). Both groups were evaluated on the baseline assessment and at the end of the 6-month MCT programme (six months after the baseline assessment). A total of 69 participants (41 TRAIN) completed the first 6-month study protocol; however, only 46 participants (30 TRAIN) had valid images from the bone assessment and enough information to be included in this study (Figure 1). Moreover, during the 6-month training period participants of both groups (CON and TRAIN) received three talks related to healthy lifestyles in order to avoid depriving the CON of positive effects.

Participants had to attend the research centre in two occasions for each evaluation. During the first day personal information and other health outcomes were collected through a structured questionnaire. Specifically, the variables included in this article were as follows: Mean of daily walking hours and sitting hours [20], smoking habits, Instrumental Activities of Daily Living Scale [21], Barthel Index [22], Mini Nutritional Assessment [23], and adherence to the Mediterranean diet [24]. Researchers performed also body composition measurements. All these measurements were performed at baseline and 6 months later. Moreover, a dietary record was obtained once, on a separate day, at the middle of the intervention timeline [25].

### 2.2. Ethics Statement

All participants received oral and written information about the aims, possible benefits, and risks derived from participation in this study. Afterwards, written informed consent was obtained from all the included participants.

The study was performed in accordance with the Helsinki Declaration of 1961 revised in Fortaleza (2013) [26] and the current legislation of human clinical research of Spain (Law 14/2007). The study protocol was approved by the Hospital Universitario Fundación de Alcorcón (16/50). This study was registered in the electronic repository *clinicaltrials.gov* (reference number: NCT03831841).

### 2.3. The EXERNET Elder 3.0 Multicomponent Exercise Program

The training protocol has been described in detail by Fernández-García et al. [27]. Exercise groups were composed of 8–16 elders depending on the space available in the training centre, and all the sessions were supervised by specialized instructors complying a maximum ratio of 12 participants per instructor (the group of 16 participants was supervised by two instructors). Sessions were held three times a week and lasted 1 h, combining exercises in order to improve endurance, strength, flexibility, balance, coordination, and functional capacity in daily life activities. All sessions included 10 min of warm-up, 35–40 min of main part exercises, and 10–15 min of cooldown. During the whole intervention period, there was a progression of the training load to ensure that the stimulus was appropriate to cause adaptations in the body. Moreover, the intensity of all exercises was adapted to each participant’s characteristics and frailty status.

### 2.4. Peripheral Quantitative Computed Tomography (pQCT)

The pQCT measurements were taken at three sites (4%, 38%, and 66%) of the tibia length using a Stratec XCT-2000 L pQCT scanner (Stratec Medizintechnik, Pforzheim, Germany). To ensure the measurements performed by the machine were correct, the pQCT device undertook a daily quality control using a validated phantom pQCT manual.

The non-dominant tibia was selected for the measurements. A coronal computed radiograph (scout view) was performed to manually locate a reference line on the distal end of the tibia. The measurement sites were located proximal to this reference line by a distance corresponding to 4% (distal tibia), 38% (diaphyseal tibia), and 66% (largest calf perimeter) of the tibia length as previously described [28]. In this study, we considered the following bone parameters: Total bone mineral content (Tt.BMC), total bone area (Tt.Ar), total bone mineral density (Tt.BMD) (all of them at 4% and 38% of the tibia length), trabecular bone density at 4% of the tibia length (Tb.BMD), cortical bone mineral density (Ct.BMD), and cortical bone thickness (Ct.Th) at 38% of the tibia length. Bone strength was established with respect to resistance to torsion (polar stress strain index in mm3 (SSIp)) and bending (measured at 38%), as fracture load X (N) (measured at 66%), with respect to the *X*-axis, as described elsewhere [29].

To ensure the quality of the measurements and obtained images, the pQCT was performed by a trained researcher (A.M.) and then the images obtained were evaluated classifying them in a scale from 1 to 5 points: 1 for those “perfect” without any movement and 5 for those “impossible to use” with too much movement during the scan test, following the indications of Blew et al. [30]. The images that scored from 4 to 5 were excluded because they were not accurate.

### 2.5. Dietary Intake

A semiquantitative food-frequency questionnaire previously validated in Spain [25,31] was used to assess the dietary intake. Previously trained researchers asked participants about their eating habits during the last year. The questionnaire included 137 items showing their typical portion size. Daily intake was calculated by multiplying the portion size by the frequency of consumption (nine options ranging from never/almost never to six or more times per day). Nutrient intake was estimated using Spanish food composition tables and other sources of information [32,33]. Nutrients estimated from the semiquantitative food frequency questionnaire and considered in this study were calcium, vitamin D, phosphorus, vitamin A (retinol), PUFA, protein, and alcohol. Reasons considered for the inclusion of these nutrients were: Calcium and vitamin D have clear evidence that they have an influence on the bone while weight adjusted protein consumption is still under debate [13]. Phosphorus and vitamin A were included because an excess of either micronutrient has shown to have a negative effect on bone metabolism. Although the National Osteoporosis Foundation only focuses its review on alcohol consumption during adolescence, we thought it would be interesting to study its role in this population as well as for PUFA, to improve the evidence of the effects of these nutrients on bone mass. Dietary reference daily intakes (RDI) were used in the study according to Spanish recommendations [32].

### 2.6. Blood Samples and Serum 25(OH)D

Fasting blood samples were collected by venepuncture (4 mL). Samples were centrifuged at 3100 rpm during 17 min for the serum extraction and they were stored at −80 °C for later analyses. To obtain 25OH-D, the “VITROS 25-OH Vitamin D Total” package was used, which includes; VITROS 25-OH Vitamin D Total Reagent Pack and VITROS 25-OH Vitamin D Total Calibrators, the VITROS ECi/ECiQ immunodiagnostic system, VITROS 3600.

### 2.7. Statistical Analysis

The Statistical Package for the Social Sciences v. 20.0 for Windows (SPSS, Inc, Chicago, IL, USA) was used to analyse the data. Normality of the sampling distribution was evaluated using Saphiro-Wilk tests. Those variables which did not follow a normal distribution were logarithmically transformed to reach the normality of variables. The statistical significance level was set at *p* < 0.05 in all tests. All the analyses were adjusted by age and sex.

Descriptive data are reported as mean and standard deviation (SD), number of participants (*n*) or percentage (%), according to the nature of each variable. Descriptive differences between groups at baseline were analysed with t-tests and chi-square tests. For those variables transformed, the original mean and SD values are reported.

Analyses of covariance (ANCOVAs) for repeated measures adjusted by age and sex were performed to analyse the group by time interactions and to compare differences in pre-training and post-training within and between groups. In addition, analyses that were repeated only included participants who assisted more than 80% of the sessions. Partial eta squared was used to report effect sizes considering the following thresholds: Small effect (>0.01), medium effect (>0.06), and large effect (>0.14). Cohen’s d was calculated for the effect size from multiple comparisons using the classic thresholds proposed by Cohen: Small effect (>0.2), medium effect (>0.5), and large effect (>0.8) [34].

Percentages of change were calculated subtracting post-intervention values minus baseline values and dividing the result by the baseline values. Then, they were multiplied by 100 to obtain a percentage. ANOVA of one factor was used to evaluate differences between groups in these variables.

Regarding dietary nutrients, individual linear regression analyses by the enter method were performed to evaluate the contribution of each nutrient variable (independent variables) on bone mass and structure percentage of changes (dependent variables), after adjusting by sex and age. If more than one nutrient showed influence on bone percentage of changes, a further step-wise regression was developed also adjusted by sex and age. Finally, similar linear regression analyses by the enter method were performed to estimate the contribution of 25(OH)D (independent variable) on bone mass and structure percentages of change (dependent variables) during training in both groups. Overall, the r^2^, r^2^ change, and standardized β are reported for all these regressions.

## 3. Results

Adherence to training averaged 85.2% ± 10.6%, ranging from 67.3% to 98.5%. There were no adverse events and no health problems due to the MCT in the TRAIN group over the 6-month intervention period. The final cohort included a total of 46 participants (16 CON (14 women) and 30 TRAIN (22 women)), which completed both evaluations of pQCT and had good quality scans. TRAIN and CON groups showed differences between height, weight, and walking hours at baseline as shown in Table 1.

### 3.1. Effects of the MCT Program on Bone Mass and Structure

Table 2 shows the group by time interactions and differences between and within groups at both evaluations adjusted by age and sex. 

Differences between groups were observed at baseline in Tt.BMC at 4%, showing better values for TRAIN (*p* < 0.05) than CON. Moreover, the same differences were observed after the 6-month MCT (*p* < 0.05).

The CON group showed decreases in Tt.BMC 4% (F_(1,41)_ = 12.297, *p* = 0.001) from 2.53 to 2.48 g (d = 0.135, small effect size) and in Tb.BMD (F_(1,2)_ = 8.435, *p* = 0.006) from 194.29 to 190.84 mg/cm^3^ (d = 0.086, small size effect). For 38% of the tibia length, decreases were observed in Tt.BMC (F_(1,36)_ = 4.278, *p* = 0.046), from 2.98 to 2.96 g (d = 0.060, small effect size), Tt.BMD (F_(1,37)_ = 10.010, *p* = 0.003), from 796.27 to 789.78 mg/cm^3^ (d = 0.064, small effect size), and in Ct.BMD (F_(1,37)_ = 6.665, *p* = 0.014) from 1128.50 to 1124.15 mg/cm^3^ (d = 0.090, small effect size).

For the TRAIN group, decreases were also observed in the same variables, with the exception of Tt.BMC at 38% of the tibia length. Concretely, there was a decrease at 4% of the tibia length Tt.BMC (F_(1,41)_ = 5.599, *p* = 0.023), from 2.84 to 2.80 g (d = 0.114, small effect size) and Tb.BMD (F_(1,42)_ = 6.875, *p* = 0.012) from 205.21 to 202.96 mg/cm^3^ (d = 0.086, small effect size). In addition, at 38% of the tibia length the decreases were found in Tt.BMD (F_(1,39)_ = 8.644, *p* = 0.006) from 810.45 to 805.93 mg/cm^3^ (d = 0.045, small effect size), and in Ct.BMD (F_(1,39)_ = 12.518, *p* = 0.014) from 1144.58 to 1139.61 mg/cm^3^ (d = 0.104, medium effect size).

No group by time interactions were observed for any variable in those previous analysis (all *p* > 0.05).

No different changes were shown when analyses were performed in those participants completing ≥80% of the training sessions.

When comparing percentages of change, differences between groups were observed for Tt.BMD at 38% of the tibia length (−0.9 ± 0.7% vs. −0.3 ± 0.5%) and Ct.Th (−0.7 ± 0.8% vs. 0.2 ± 1.1%); both *p* < 0.05, CON and TRAIN, respectively. All percentages of change are presented in Table 3.

### 3.2. Dietary Intake Descriptive Variables and Their Contribution to Changes in Bone Mass and Structure during the 6-Month Intervention Period

Table 4 shows the intake of bone-related nutrients included in this report and their RDI.

No differences of consumption were observed between groups for any nutrient (all *p* > 0.05). The mean intake of our cohort was above the recommended levels for dietary vitamin A and phosphorus, but it did not reach the excess threshold. The intake levels for the other nutrients were below recommendations.

As shown in Table 5, dietary vitamin A negatively influenced changes in Tt.BMD at 4%, Tt.BMD and Ct.BMD at 38% of the tibia length, showing detrimental effects with predictive values of 9.2%, 17.4%, and 15.8%, respectively after taking sex and age into account. 

Dietary PUFA were positively associated with changes showing benefits for Tt.BMD at 4% (change r^2^ = 0.097). Moreover, for changes in Fracture load, alcohol seems to be harmful showing a negative influence (change r^2^ = 0.149) (all *p* < 0.05).

When dietary vitamin A and PUFA were included in a step-wise regression analysis with Tt.BMD as the independent variable, dietary vitamin A seemed to decrease Tt.BMD (change r^2^ = 0.174, β standardized = −0.425) and the influence of PUFA disappeared.

No contribution to bone changes were found in the model for dietary P, Ca, dietary vitamin D nor protein.

When serum 25(OH)D was analysed as a predictive variable for bone percentage of change (also adjusted for sex and age), the only significant outcome was observed for Ct.BMD at 38% of the tibia length (overall r^2^ = 0.233, change in r^2^ = 0.081, and β unstandardized = 0.014). No other bone analysed variable was statistically significant (all *p* > 0.05).

## 4. Discussion

The main findings of this study are: (1) MCT seems to have a minor but significant impact on bone variables by the attenuation of some decreases related to aging; (2) alcohol intake and an excess of dietary vitamin A have a negative effect on bone changes while PUFA seems to prevent the bone decline, (3) serum levels of 25(OH)D showed a small positive association with changes on Ct.BMD.

Bone metabolism across the lifespan has been widely studied. With age, there is a decrease not only in Tt.BMD, but also in cortical and trabecular bone, with a consequent weakening of bone structure [35]. Although Dual Energy X-ray Absorptiometry is the gold-standard and the most widespread technique in intervention studies, it is not able to evaluate volumetric variations of the bone. Thus, pQCT has been recommended in order to study bone changes with more precision.

In this regard, the effects of MCT on the bone still remain under debate [12] and there is no evidence until now about the effects of this type of intervention on bone structure in frail or pre-frail older adults. In our cohort, decreases were observed after the 6-month intervention period in Tt.BMC and Tb.BMD at 4% and in Tt.BMD and Ct.BMD at 38% of the tibia length, independently of the group (CON or TRAIN). Moreover, the CON group showed a decrease in Tt.BMC at 38% of the tibia length. When these changes between groups were studied, those who exercised presented smaller losses in Tt.BMD at 38% of the tibia length and in Ct.Th when compared to CON.

Even MCT seems to be effective improving physical fitness [5], this MCT may not be the best protocol for the improvement of bone mass, although it seems useful to slightly minimize the detrimental effect of ageing in bone in pre-frail and frail elders. The lack of clearer results may be partially explained due to the TRAIN group showing greater baseline values, which could lead to a higher probability of losses and also to the small statistical power related with our small cohort. Moreover, CON showed more walking hours than TRAIN, which could also be delaying the decline. Other effects of our MCT on other variables which are particularly important in osteoporosis, such as balance, risk of falls, and fracture, should be studied in future.

As it has been stated above, there is some controversy about the efficacy of MCT in bone mass of elderly people. For instance, Tolomio et al. found increases in the femoral neck after an 11-week intervention [36], while Karinkanta et al. found smaller negative changes in the MCT group when compared to other exercise types in SSIp [37], and Marín-Cascales did not find changes [38]. However, MCT is a generic term referring to exercise programs which involves strength and endurance exercises overall but also another type of exercise such as stretching or balance. MCT design could differ among each practitioner, physical educator, or trainer depending on their objectives, thus, large differences among exercise protocols or characteristics of the study cohort (such as nutritional status) may explain discrepancies between studies, which does not allow drawing a conclusion about the MCT effects on bone parameters.

Another important difficulty when we aim to interpret the effects of training protocols on bone health is the bone-nutrition-exercise interaction that has been previously described in other populations [39,40]; and that is generally little taken into account in these types of studies that describe training effects. In fact, given the burden of osteoporosis in our aging population, well designed control trials have been suggested to be timely and much needed [41]. In this sense, it is known that bone metabolism is complex, and highly influenced by lifestyle habits, among which is dietary intake [9]. For this reason, the nutritional status of participants may play an important role in the effects of exercise programs on bone mass and structure. In our study, alcohol intake showed negative associations with fracture load explaining up to 14.9% of the variability. Daily alcohol intake, specifically wine, has been promoted as part of the healthy Mediterranean Diet [24] and this is one of the reasons why it is habitual in the Spanish older population. However, detrimental associations with an increased risk of some diseases such as cancer [42] or osteopenia [43] have been well described. Its consumption could be compromising the absorption of nutrients and it could be reducing exercise effects, so it should not be encouraged in this population.

Other dietary nutrients that were related to bone changes in our results were dietary PUFA and vitamin A. Specifically, PUFA accounted for up to 9.8% of the changes found at Tt.BMD at 4% of the tibia length, which means that it could have a beneficial effect on bone health. Although this micronutrient has been less studied, a possible explanation could be that it plays a role in inflammation, inhibiting osteoclastic activity by complex pathways described elsewhere [44,45]. Otherwise, in the present study, it has been observed that dietary vitamin A has been also associated to higher decreases in bone parameters; vitamin A is a potential antioxidant with multiple benefits in the ageing process but which can produce breakdown effects in the bone if it is consumed in an excess [46]. The mean intake of our cohort was above the recommended levels, but it did not reach the excess threshold. However, vitamin A was negatively associated with Tt.BMD at 4% and at 38% of the tibia length; and Ct.BMD accounting for up to 17.4% of the total variability. It will be interesting to consider food sources in order to study these negative associations, as vitamin A can come from vegetables or animal foods and could have a different influence on bone health [47,48]. However, more accurate studies are needed, with larger cohorts, to analyse those nutrients with lower levels of evidence, such as vitamin A or PUFA.

Finally, although evidence regarding the relation of bone parameters with dietary calcium, vitamin D from diet, and serum 25(OH)D have been defined as strong [13], no associations were found between these dietary intake reported variables and bone changes during the 6-month intervention period. As mean intakes of dietary calcium and vitamin D of both groups were close to the recommendations, a possible influence in bone changes could be hidden. However, when blood serum 25(OH)D was evaluated, a slightly positive association was observed with cortical bone thickness. Moreover, Uusi et al. reported an increase in trabecular BMD in the vitamin D supplemented and exercise group but not in the exercise non supplemented group [49]. Nevertheless, when this intervention stopped none of the exercises, vitamin D, or combined groups reduced the normal bone ageing decline [50]. Thus, more studies are needed to establish clear associations, as supplementation seems to be effective [49], adequate values of serum 25(OH)D have previously been associated with muscle gains [14] and its deficiency with frailty status [51].

Some limitations of this study should be mentioned. Even though, to the best of our knowledge, our study is the first one evaluating the effects of MCT in bone mass and structure of frail and pre-frail elders, the sample size is small, which could lead to a lack of statistical power. Even randomized clinical trials are recommended, to ensure that participation and attendance of this population is very difficult and thus we allocate them by convenience. Greater samples should be recruited in order to study the differences between frail and pre-frail elders. Despite the fact that a large number of cohort images could not be included because of movement during testing, the use of pQCT should be highlighted as an important strength, which allows differentiating the bone structure and the use of a quality image scale is another strength, which improves the quality of the measurements. Reasons found for these movements were: difficulty to stand still during the assessment, to have trembles due to medications, and to be nervous because of the evaluation. Thus, bigger samples should be included while expecting this big loss including a more relaxed atmosphere for testing. In addition, while it was difficult to encourage attendance for CON, no one in TRAIN revoked their participation. Moreover, all participants have requested to participate again in a new phase of the project. Thus, MCT is an effective strategy not only for slightly reducing bone detriment but also to increase exercise adherence in this population. Other limitations derived from the self-report and FFQs should be mentioned as older people could be under- or over- estimating their food consumption. Finally, the lack of information about the presence of any treatment with bone-active agents should be considered as a limitation of this study, as it may hinder the real effects of MTC on bone variables.

## 5. Conclusions

To sum up, the MCT program seems to slow down some of the bone reductions associated with ageing in frail and pre-frail older adults; however, we could not recommend this type of intervention as there should be other protocols more optimized to improve the bone structure in this population. Serum 25(OH)D seems to have a limited positive influence on bone changes. In addition, dietary nutrients such as alcohol or vitamin A seem to have a negative effect on changes of fracture index, and BMD variables, but more studies are needed in order to explain the interrelation of other nutrients. The authors recommend avoiding alcohol intake and having an adequate consumption of food sources with vitamin A intake in order to protect the bone health.

## Figures and Tables

**Table 1:** Descriptive characteristics of the cohort at baseline.

	CON (*n* = 16)	TRAIN (*n* = 30)	*p*-Value
**Age and Anthropometrics**			
	**Mean ± SD**	**Mean** ± **SD**	
Age (years)	79.1 ± 5.1	81.3 ± 5.0	0.162
Height (cm)	153.1 ± 7.2	159.3 ± 8.1	**0.037**
Weight (kg)	67.0 ± 7.2	73.8 ± 11.3	**0.046**
BMI (kg/m^2^)	29.2 ± 4.3	29.3 ± 3.6	0.952
**Walking hours per day**	2.1 ± 1.5	1.0 ± 0.8	**0.014**
**Sedentary hours per day**	5.9 ± 2.8	6.1 ± 2.6	0.907
**Sex**	***n* (%)**	***n* (%)**	
Women	14 (87.5)	22 (73.3)	0.267
Men	2 (12.5)	8 (26.7)	
**SPPB**			
Frail	1 (6.3)	5 (16.7)	0.312
Pre-frail	15 (93.7)	25 (83.3)	
**Health related habits**			
Smoke			
Yes	2 (12.5)	0 (0.0)	0.220
No	14 (87.5)	30 (100.0)	
**Nutrition related questionnaires**			
ADM			
Yes	11 (68.7)	23 (76.7)	0.827
No	5 (31.3)	7 (23.3)	
MNA			
At risk of malnourishment	1 (6.3)	6 (20.0)	0.291
Normal nutritional status	15 (93.7)	24 (80.0)	
**Dependence questionnaires**			
Barthel Index			
Mildly dependendent	7 (43.7)	9 (30.0)	0.348
Independent	9 (56.3)	21 (70.0)	
IADL			
Moderate dependence	3 (18.2)	4 (13.3)	0.220
Mildly dependendent	1 (6.3)	9 (30.0)	
Independent	12 (7.5)	17 (56.7)	

CON: Control group; TRAIN: Training group; BMI: Body Mass Index; SPPB: Short Physical Performance Battery; ADM: Adherence to Mediterranean Diet; MNA: Mini Nutritional Assessment; IADL: Instrumental activities for daily living. Bold letters represent statistical significance *p* < 0.05.

**Table 2:** Bone variables in CON and TRAIN groups at pre-training and post-training periods adjusted by age and sex.

	CON			TRAIN			
	Pre-Training	Post-Training	F	Pre-Training	Post-Training	F	GxT Interaction η_p_^2^
Tt.BMC 4% (g)	2.53 ± 0.37	2.48 ± 0.37	12.297 *	2.84 ± 0.35 ^♦^	2.80 ± 0.35 ^♦^	5.599 *	0.046
Tt.BMD 4% (mg/cm^3^)	233.50 ± 40.24	232.68 ± 41.66	0.149	250.78 ± 39.81	251.37 ± 41.19	0.151	0.007
Tb.BMD 4% (mg/cm^3^)	194.29 ± 39.46	190.84 ± 40.52	8.435 *	205.21 ± 39.04	202.96 ± 40.09	6.875 *	0.015
Tt.Ar 4% (mm^2^)	6.99 ± 0.03	6.97 ± 0.03	2.060	7.03 ± 0.02	7.01 ± 0.02	2.271	0.002
Tt.BMC 38% (g)	2.98 ± 0.33	2.96 ± 0.33	4.278 *	3.14 ± 0.32	3.13 ± 0.32	1.932	0.017
Tt.BMD 38% (mg/cm^3^)	796.27 ± 99.97	789.78 ± 100.66	10.110 *	810.45 ± 98.66	805.93 ± 99.34	8.644 *	0.015
Ct.BMD 38% (mg/cm^3^)	1128.50 ± 47.80	1124.15 ± 47.82	6.665 *	1144.58 ± 47.35	1139.61 ± 47.38	12.518 *	0.001
Ct.Th 38% (mm)	4.28 ± 0.63	4.25 ± 0.63	3.289	4.47 ± 0.62	4.47 ± 0.62	0.117	0.039
Tot.Ar 38% (mm^2^)	5.92 ± 0.02	5.93 ± 0.02	1.418	5.95 ± 0.02	5.95 ± 0.02	0.120	0.014
Fracture Load X 38% (N)	5385.55 ± 771.14	5290.65 ± 855.09	0.335	5665.81 ± 762.35	5552.71 ± 845.33	2.088	0.003
SSIp 38% (mm^3^)	1385.07 ± 190.08	1374.73 ± 195.66	0.736	1478.21 ± 187.59	1476.46 ± 193.14	0.006	0.014

CON: Control group; TRAIN: Training group; Tt.BMC: Total bone content; Tt.BMD: Total bone density; Tb.BMD: Trabecular bone density; Tt.Ar: Total bone area; Ct.BMD: Cortical bone density; Ct.Th: Cortical bone thickness; SSIp: Polar stress strain index; GxT: Group by time. * Statistical significance differences within groups between pre-training and post-training, *p* < 0.05. ^♦^ Statistical significance differences between groups at pre-intervention or post-intervention *p* < 0.05.

**Table 3:** Differences in percentages of change of bone-related variables between CON and TRAIN groups.

	CON	TRAIN	*p*-Value
Tt.BMC 4% (g)	−2.4 ± 3.2	−1.0 ± 1.9	0.089
Tt.BMD 4% (mg/cm^3^)	−0.3 ± 3.8	0.1 ± 2.1	0.799
Tb.BMD 4% (mg/cm^3^)	−1.9 ± 2.4	−1.2 ± 2.4	0.360
Tot.Ar 4% (mm^2^)	−1.9 ± 6.1	−0.9 ± 3.0	0.496
Tt.BMC 38% (g)	−0.6 ± 0.9	−0.3 ± 1.3	0.572
Tt.BMD 38% (mg/cm^3^)	**−0.9 ± 0.7**	**−0.3 ± 0.5**	**0.006**
Ct.BMD 38% (mg/cm^3^)	−0.5 ± 0.6	−0.3 ± 0.5	0.463
Ct.Th 38% (mm)	**−0.7 ± 0.8**	**0.2 ± 1.1**	**0.012**
Tot.Ar 38% (mm^2^)	0.4 ± 0.9	0.0 ± 1.1	0.302
Fracture Load X 38% (N)	−1.3 ± 5.9	−2.2 ± 6.8	0.731
SSIp 38% (mm^3^)	−0.1 ± 2.3	−0.1 ± 3.9	0.999

CON: Control group; TRAIN: Training group; Tt.BMC: Total bone content; Tt.BMD: Total bone density; Tb.BMD: Trabecular bone density; Tt.Ar: Total bone area; Ct.BMD: Cortical bone density; Ct.Th: Cortical bone thickness; SSIp: Polar stress strain index. Bold letters represent statistical significance set at *p* < 0.05.

**Table 4:** Descriptive dietary intake variables per day from the semiquantitative food frequency questionnaire, serum 25(OH)D, and adherence to Spanish recommendations.

		CON	TRAIN	*p*-Value
Protein (g/kg)		1.38 ± 0.28	1.42 ± 0.40	0.743
Alcohol (g)		1.10 ± 1.64	1.16 ± 1.87	0.744
PUFA (g)		15.94 ± 7.11	20.23 ± 9.38	0.134
**Vitamins**	**RDI (F/M)**			
Vit A (µg)	800/1000	1480.26 ± 765.08	1583.69 ± 504.71	0.601
Vit D (µg)	20	5.65 ± 3.40	5.95 ± 3.15	0.774
**Minerals**				
Ca (mg)	1200	994.86 ± 266.71	1176.68 ± 370.07	0.102
P (mg)	700	1709.05 ± 393.83	1898.33 ± 444.44	0.177
**25(OH)D**	**Normal Values**			
Vit D (ng/dL)	>30	24.17 ± 3.28	27.32 ± 10.71	0.392

CON: Control group; TRAIN: Training group; PUFA: Polyunsaturated fatty acids; Vit A: Vitamin A; Vit D: Vitamin D; Ca: Calcium; P: Phosphorus; 25(OH)D: Serum 25-hydroxyvitamin D; RDI: Recommended daily intake; F: Female; M: Male. Statistical significance set at *p* < 0.05.

**Table 5:** Sex- and age- adjusted linear regression coefficients for the influence of dietary intake on bone mass and structure.

		Tt.BMC 4% (g)	Tt.BMD 4% (mg/cm^3^)	Tb.BMD 4% (mg/cm^3^)	Tt.Ar 4% (mm^2^)	Tt.BMC 38% (g)	Tt.BMD 38% (mg/cm^3^)	Ct.BMD 38% (mg/cm^3^)	Ct.Th 38% (mm)	Tt.Ar 38% (mm^2^)	Fracture Load X 38% (N)	SSIp 38% (mm^3^)
	Overall r^2 b^	0.176	0.041	0.031	0.047	0.052	0.062	0.229	0.042	0.057	0.138	0.195
Calcium (mg)	Change r^2^	0.000	0.014	0.002	0.004	0.004	0.011	0.027	0.005	0.013	0.001	0.067
	Standardized β	0.008	0.123	0.052	0.711	−0.062	0.110	0.170	−0.073	−0.120	−0.033	−0.269
Vitamin D (µg)	Change r^2^	0.079	0.036	0.085	0.063	0.005	0.001	0.008	0.009	0.004	0.009	0.000
	Standardized β	0.288	−0.193	0.298	0.256	−0.070	0.023	0.090	−0.100	−0.062	0.096	−0.020
Vitamin A (µg)	Change r^2^	0.035	**0.092**	0.065	0.079	0.016	**0.174**	**0.158**	0.001	0.011	0.051	0.003
	Standardized β	0.189	**−0.306**	0.256	0.283	−0.131	**−0.425**	**−0.405**	−0.030	0.109	0.227	−0.052
Phosphorus (mg)	Change r^2^	0.000	0.003	0.024	0.000	0.021	0.001	0.021	0.045	0.022	0.001	0.036
	Standardized β	0.019	0.055	0.164	−0.010	−0.150	0.026	0.150	−0.220	−0.155	0.033	−0.197
Protein (g/kg)	Change r^2^	0.008	0.042	0.003	0.006	0.072	0.001	0.019	0.077	0.060	0.033	0.057
	Standardized β	0.087	0.205	−0.050	0.079	−0.269	−0.025	0.134	−0.278	−0.252	−0.188	−0.241
PUFA (g)	Change r^2^	0.003	**0.097**	0.005	0.091	0.085	0.028	0.001	0.062	0.061	0.021	0.002
	Standardized β	−0.055	**0.339**	0.074	−0.236	0.317	0.181	−0.042	0.270	0.268	−0.156	0.045
Alcohol (g)	Change r^2^	0.016	0.016	0.021	.026	0.033	0.104	0.024	0.030	0.000	**0.149**	0.002
	Standardized β	−0.137	0.137	−0.156	−0.176	0.207	0.368	0.177	0.194	0.014	**−0.466**	−0.054

Tt.BMC: Total bone content; Tt.BMD: Total bone density; Tb.BMD: Bone trabecular density; Tt.Ar: Total bone area Ct.BMD: Cortical bone density; Ct.Th: Cortical bone thickness; SSIp: Polar stress strain index; PUFA: Polyunsaturated fatty acid. Standardized β and change in r^2^ for each of the nutrient intake predictors adjusted by age and sex. ^b^ From the age and sex prediction model. Characters in bold indicate statistical significance (*p* < 0.05).
